# Crohn's disease of the vulva: A tough diagnosis (a case report of a 47 y.o. patient)

**DOI:** 10.1002/ccr3.2707

**Published:** 2020-02-06

**Authors:** Karina R. Bondarenko, Yulia E. Dobrokhotova, Tatiana A. Rumyantseva, Nailya I. Nasyrova

**Affiliations:** ^1^ Department of Obstetrics and Gynecology Therapeutic Faculty Pirogov Russian National Research Medical University Moscow Russia; ^2^ Clinical Diagnostic Center Central Research Institute of Epidemiology Moscow Russia; ^3^ Department of Gynecology Central Clinical Hospital of Civil Aviation Moscow Russia

**Keywords:** hypertrophy of the vulva, vaginal discharge, vaginal infection, vulvar Crohn's disease

## Abstract

Extraintestinal symptoms of Crohn's disease may occur in the eye, the urinary system, on the skin, or subcutaneous fat. We report the case of a 7‐year diagnostic search of isolated vulvar Crohn's disease in a 47‐year‐old woman. The disease is characterized by gradual formation of pronounced bilateral asymmetric labial hypertrophy.

## Graphical abstract

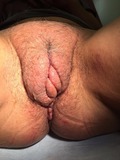

## 1 INTRODUCTION

Crohn's disease (CD) is a chronic inflammatory granulomatous disease of the gastrointestinal tract affecting almost any part of it from the oral cavity to the rectum.^1^ The inflammation in CD is usually transmural and segmental, which in some cases causes the involvement of other parts of the intestine and adjacent organs (eg, genitalia) accompanied by fistulas and abscesses.^2^ Extraintestinal manifestations of CD are associated mainly with direct (through the fistulas) or metastatic spread of inflammation from the primary focus.^3^ However, an isolated form of CD may have an extraintestinal localization in the perineal region, affecting mainly the vulva, with no pathomorphological features typical of CD in the intestines (CD of the vulva).^3^ Epidemiological data on the prevalence of CD of the vulva are currently scarce, which is probably due to misdiagnosis. In some cases, it took patients 5 years to be diagnosed with CD of the vulva.^4^ To date, to the best of our knowledge, 146 cases of isolated CD of the vulva have been described worldwide.^5^ Thus, reports on each confirmed case of CD of the vulva contributes to the accumulation of international knowledge and experience in the field and, which can significantly shorten the time between manifestation of the first symptoms and the correct diagnosis. The case of CD of the vulva in a 47‐year‐old woman is reported.

## 2 CASE REPORT

A 47‐year‐old white female complained of a recurrent severe vulvar edema, itching, excessive vaginal discharge, fever (37.5°C), heaviness in the genital area, and painful intercourse.

The patient had 3 pregnancies, 2 deliveries, 1 abortion. She menstruated regularly. She had first sexual intercourse at the age of 16. The patient was married and had regular sexual intercourse with one partner, contraception was coitus interruptus.

Previously registered gynecological morbidities included several episodes of vulvovaginal candidiasis, bacterial vaginosis, uterine leiomyomas, and endometrial polyp.

The first symptoms of the disease developed 7 years before after heavy insolation. The symptoms included fever (38.5°C), itching in the pubic region, prominent edema, hyperemia of the vulva and perianal region, vulvar induration, difficulty urinating, homogeneous white vaginal discharge, vulvar fissures, and enlargement of the inguinal lymph nodes. The sequence of the symptoms remained stable for 7 years: itching, followed by edema and hyperemia of the external genitalia within 2‐3 hours, followed by fever. Topical and systemic antibacterial and symptomatic therapies were chosen (vuvlovaginal infection was suspected). The symptoms recurred a few weeks after the onset. The second episode occurred 6 months later, without any specific reason. Symptomatic treatment was chosen, the symptoms gradually relieved. The further course of the disease was characterized by the reduction of the remission period from 6 to 2‐3 months with less severe manifestations during each recurrence, which was apparently due to the immediate start of therapy, including local and systemic glucocorticoids and antimicrobials (with no diagnosis). Phases of the menstrual cycle, coitus, infectious diseases, medications, food, and other did not influence the severity and frequency of relapses. Later certain disease manifestations were observed even between recurrences, namely moderate hypertrophy and induration of the labia minora and labia majora, which gradually increased in size as the disease progressed.

Nonspecific inflammatory markers were found in the patient's laboratory tests (Table 1). The immunogram, the biochemical blood test, and the coagulogram were within reference values. The level of the total serum IgE did not exceed standard values. There was an increase in the antistreptolysin‐O concentration with a normal rheumatoid factor. Of the autoimmune disease markers, antinuclear antibodies were increased, with SS‐A antibodies being present.

**Table 1:** Biochemical and immunological blood test results

Parameters	Result	Reference interval
Biochemistry		
RF, IU/mL	5.8	<14
ASLO, IU/mL	562	<200
C‐reactive protein, mg/L	2.4	<5
Immunological parameters		
Total IgE, U/mL	72.5	<100
C3 complement component, g/L	1.16	<1.85
C4 complement component, g/L	0.159	<0.53
Autoimmune markers		
Anticardiolipin antibodies (IgG, IgM, IgA), IU/L	2.2	0‐12.0
Antinuclear antibodies (quant. IgG), positivity coefficient	4.0 ++	<1.0 not found 1.0‐1.2 controversial result >1.2 found
Sm, RNP/Sm, SS‐A (60 kDA), SS‐B, Scl‐70, PM‐Scl, PCNA, CENP‐B, dsDNA, Histone, Nucleosome, Rib P, AMA‐M2, Jo‐1 antigen	Not found	Not found
SS‐A (52 kDa)	+++	Not found
Antibodies against double‐stranded DNA, (quant. IgG), IU/mL	5.2	<20
Antinucleosomic antibodies, (quant. IgG), IU/mL	10.3	<15
Anticentromeric antibodies, (quant. IgG), IU/mL	1.6	<10
Antispermal antibodies, (quant. IgG), IU/mL	41.9	<55

Brush cytology from the affected site revealed dermatitis, unspecified. Colonoscopy (Olympus CF type H180AL) did not reveal significant structural changes in the intestines and anal condylomas. Esophagogastroduodenoscopy (Olympus GIF H180J) showed sporadic hemorrhagic erosions associated with gastritis, focal bulbitis, and distal catarrhal esophagitis along with cardiac insufficiency. Histologically, gastritis with hyperplasia of gastric epithelium was confirmed.

Despite the fact that the patient was followed up by different specialists, including a gynecologist, a dermatologist, an immunologist, a gastroenterologist, a rheumatologist, a proctologist, an infectious disease specialist, an urologist, a psychotherapist, and other specialists, CD of the vulva was not suspected for 7 years. The patient sought medical advice 10 times yearly on average and was examined and treated twice at a DV clinic. Since the infectious and/or autoimmune etiology of the disease was suspected, the search for infectious pathogens, inflammatory and autoimmune disease markers was a priority. However, no antibodies against *Treponema pallidum*, HIV, Hepatitis B and C viruses, as well as protozoan infections (trichinella, toxocara, echinococci) were detected. Every year, vaginal and cervical samples were examined by microscopy, cultures, and PCRs for *Neisseria gonorrhoeae*, *Chlamydia trachomatis*, *Mycoplasma genitalium*, *Trichomonas vaginalis*, *Gardnerella vaginalis*, *Candida albicans*, *Ureaplasma parvum*, *Ureaplasma urealyticum*, *Mycoplasma hominis*, *HPV*, *HSV*, and *CMV.* STIs were never detected by any method. The sample was also tested for the presence of *M tuberculosis*, *Candida* spp., and *Actinomyces* DNA; the results were negative. Repetitive antimicrobial treatment was administered due to lactobacillus depletion or *Streptococcus agalactiae* detection.

Medications used to treat the disease included gentamycin, ampicillin, amoxicillin/clavulanic acid, ceftriaxone, ceftibuten, levofloxacin, moxifloxacin, doxycycline, nifuratel, antiviral drugs (valacyclovir), probiotics and immunomodulators, parenteral and oral glucocorticoids, nonsteroidal anti‐inflammatory drugs, and antihistamines. Topical therapy included numerous ointments, gels, creams, vaginal suppositories, tablets containing antiseptics, glucocorticoids, substances increasing regeneration and metabolism, vitamins, and probiotics. Remission was achieved only when systemic or topical glucocorticoids and, in certain cases, antibiotics were used. The patient underwent three courses of plasmapheresis; after the third procedure, her general condition deteriorated, the body temperature elevated to 37.8°C; vulvar edema and hyperemia accompanied by exacerbated induration, which caused discontinuation of plasmapheresis.

## 3 DIFFERENTIAL DIAGNOSIS, INVESTIGATIONS, AND TREATMENT

**Figure 1:**
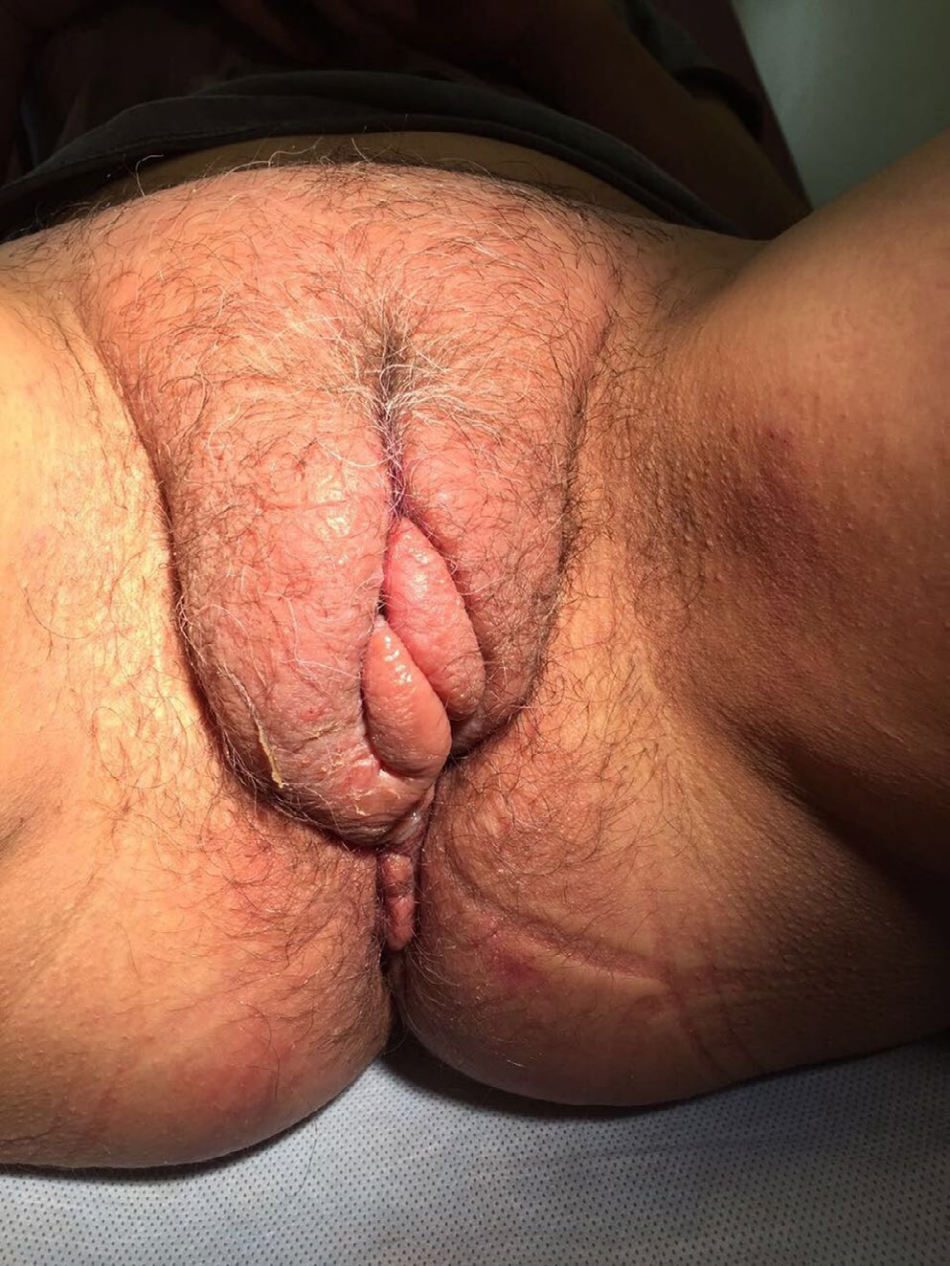
Bilateral asymmetric hypertrophy of the vulva in Patient K. with vulvar Crohn's disease

Gynecological examination (Figure 1) revealed bilateral asymmetric hypertrophy, prominent edema of the labia majora, predominantly on the right side. Fissures with clear liquid discharge, in certain places covered with crusts, were seen on the surface of the labia majora. The labia majora were extremely hard (indurative edema), partially flexible, and painless. The labia minora were dense at both sides and increased in size. Due to hypertrophy, they were not covered by the labia majora and asymmetrically protruded beyond the edges of the latter; the edges of the labia minora being slightly coarse with papilliform vegetations. The anatomy of the urethral external meatus and clitoris was not changed. No signs of inflammation of the vagina and the cervix were registered; vaginal discharge was excessive, white in color, and homogenous. Vaginal swabs were examined by microscopy and PCR. Bimanual examination did not reveal any abnormalities.

**Figure 2:**
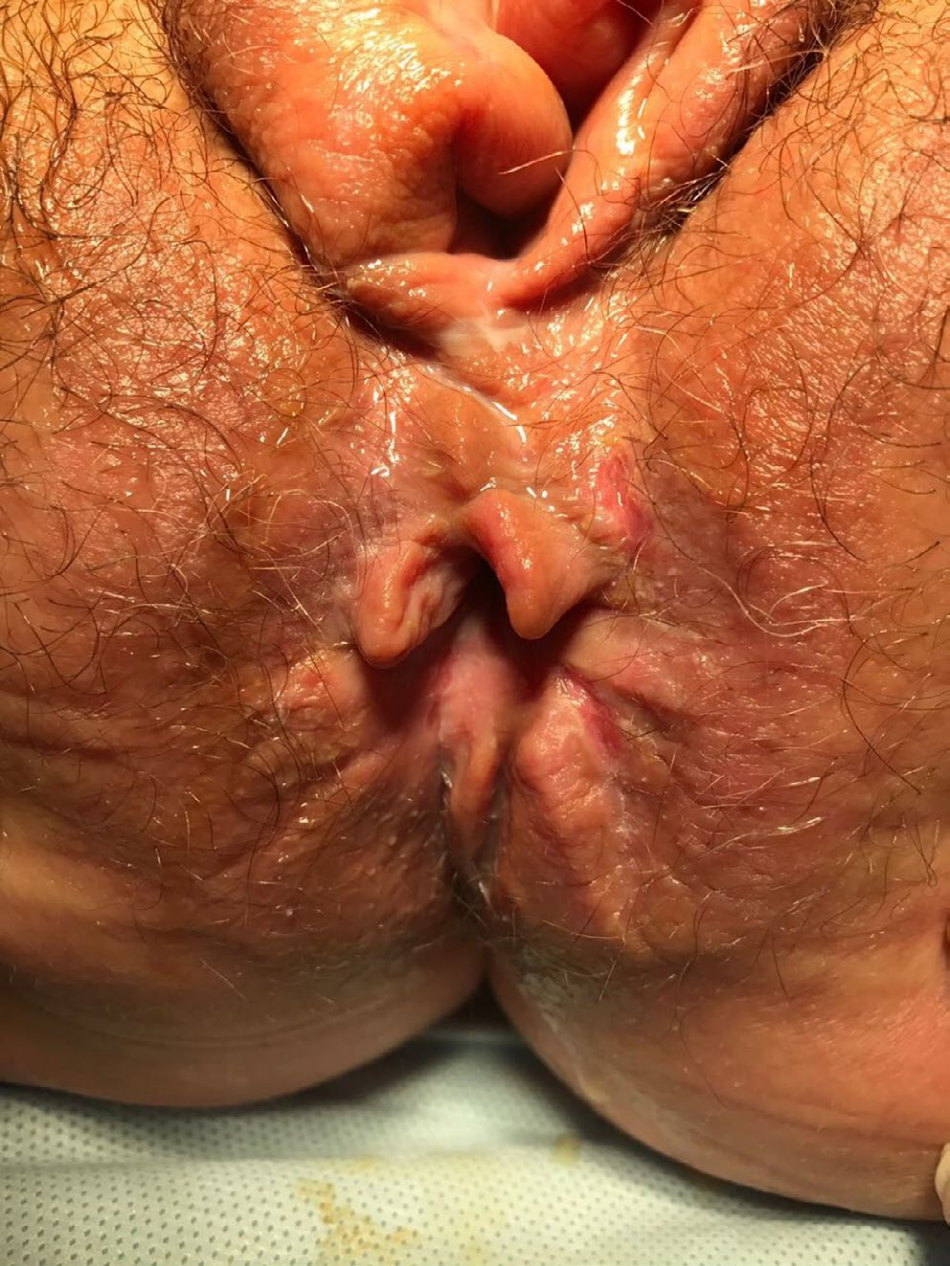
Cone‐shaped vegetations resembling condyloma acuminata in the perianal region of the vulva in Patient K. with vulvar Crohn's disease

Cone‐shaped vegetations resembling condyloma acuminate and hyperemia were observed in the perianal region (Figure 2).

Ultrasound examination revealed lymphadenitis (bilateral enlargement of lymph nodes up to 3 cm).

On the day following the visit, itchy erythematous rash appeared on the back surface of the patient's hands and the anterior surface of the thighs. After a single injection of betamethasone, the rash subsided over 4 days.

Differential diagnosis of CD, sarcoidosis, vulvar cancer, tuberculosis, actinomycosis, and lymphogranuloma venereum was made.

The CBC, biochemical, and hemostasiological parameters were within normal ranges.

The increased antinuclear factor was repeatedly detected, the presence of SS‐A antibodies was confirmed as well. The level of antibodies against *Saccharomyces cerevisiae* (ASCA) considered to be the laboratory marker of CD was slightly elevated to 21.31 rel. μ/mL (reference value <20 rel. μ/mL).

**Figure 3:**
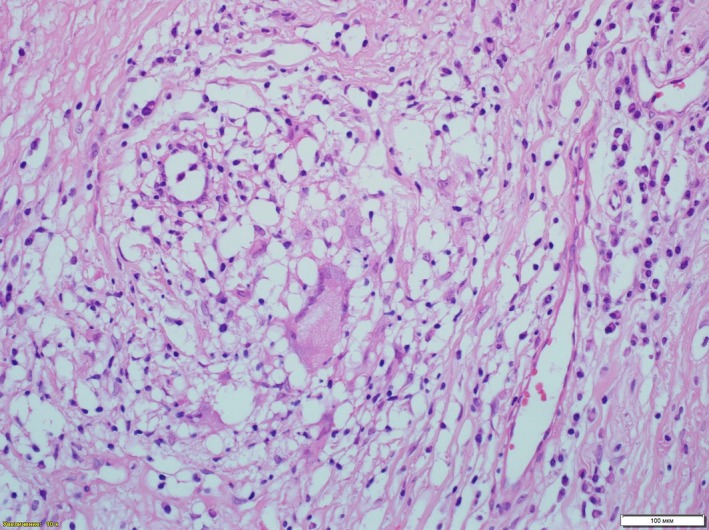
Deeper dermis shows small and large lymphoid aggregations and nonnecrotizing ("noncaseous") histiocytic granulomas with epithelioid cells and Langhans giant multinuclear cells (H and E, ×200)

The biopsy of the affected region (the central portion of the right labium majora, and the most hypertrophic portion of the left labium minora) was performed (depth of 1‐1.5 cm). The biopsy revealed pathomorphological signs of CD. Orthokeratosis, hyperkeratosis, and focal acanthosis were seen on the surface of the samples (Figure 3). Edema, lymphangiectasia, dilated vessels, histiocytic and lymphoid cells with focal perivascular lymphohistiocytic infiltration with microgranuloma formation, the signs of obliterating granulomatous lymphangitis, and lymphoplasmacytic infiltration were detected in derma (Figure 3). Small and large lymphoid aggregations and nonnecrotizing ("noncaseous") histiocytic granulomas with epithelioid cells and Langhans giant multinuclear cells were also registered. In addition, signs of neoangiogenesis, proliferating nerve fibers with focal signs of neuritis and sclerotic areas were visualized.

Computer tomography of the abdomen was performed (General Electric Medical Systems Optima CT66), with no pathological findings (fistulas, abscesses, masses) in the lungs, digestive, urinary, and reproductive systems.

Colonoscopy (Olympus CF‐HQ 150) did not reveal any endoscopic signs of the disease of the intestine mucosa.

## 4 DISCUSSION

Crohn's disease is known to cause abdominal pain, diarrhea, and body weight loss.^1^, ^2^ In some cases, extraintestinal symptoms may occur in the gastrointestinal tract (primary sclerosing cholangitis, cholangiocarcinoma, pericholangitis, fatty hepatosis, chronic hepatitis), in the eye (iritis, episcleritis, keratitis, conjunctivitis, blepharitis), joints (polyarthritis, spondylitis, arthralgia), the urinary system (pyelonephritis, nephrolithiasis), blood (autoimmune hemolytic anemia, iron‐ and B_12_‐deficiency anemia), as well as on the skin and subcutaneous fat (nodular erythema, gangrenous pyodermia, lower limb ulcers, anal pruritus, dermatitis, abscesses, phlegmonas).^1^, ^2^ Cutaneous manifestations of CD are reported to precede the appearance of gastrointestinal symptoms in 25% of cases.^3^ Unfortunately, available data do not provide a detailed description of clinical manifestation characteristics, diagnostic algorithms, and options of CD treatment with the predominant lesions localization in the perineal skin region without any specific involvement of the intestines (CD of the vulva). Since the first description of extraintestinal vulvar CD in 1965,^6^ the total number of verified diagnoses in the world has amounted to several dozens,^3^, ^7^, ^8^ so the possibility that an individual specialist may come across a patient with this disease being extremely low. Thus, the prevalence of isolated vulvar CD may be higher than described but rarely diagnosed. The variability of the symptoms and characteristics of the course of vulvar CD also plays part in a considerable delay in the diagnosis verification.^3^, ^6^, ^9^ Cutaneous signs of vulvar CD normally include perineal edema and erythema accompanied by itching and/or pain, which, with further progression of the disease, are transformed into unilateral vulvar hypertrophy, abscesses, and ulcers with fistulas.^3^, ^8^, ^10^, ^11^ Ulcerations in the inguinal folds resembling knife cuts are considered to be a pathognomonic symptom of vulvar CD.^3^, ^8^, ^10^, ^11^ The dynamics of Patient K.'s disease virtually corresponded to the stages of development of vulvar CD described in the literature. The absence of abscesses and fistulas likely due to aggressive long‐term antibacterial therapy for the suspected reproductive tract infection and a lesser degree of skin damage with no formation of “knife cuts” typical of vulvar CD are specific features of the disease in this case. It is important to note that today there are no laboratory CD predictors to suggest the development of the disease with high accuracy. Changes in blood tests are nonspecific; the development of CD is usually not accompanied by any specific autoimmune markers. Vulvar CD can be diagnosed after ruling out all infectious and noninfectious diseases with similar clinical manifestations, such as actinomycosis, sarcoidosis, tuberculosis, candida infection, herpes virus infection, schistosomiasis, lymphogranuloma venereum, chronic lymphadenitis, cellulitis, hidradenitis, a foreign body, and neoplasia.^9^, ^10^, ^12^ Granulomatous inflammation without caseous necrosis is the main histological feature of CD, including its vulvar form.^1^, ^2^, ^3^ This case report demonstrates that brush cytology of the damaged area surface was insufficient for morphological verification of vulvar CD. Biopsy of the affected areas is strongly recommended for verification.

Endoscopic examinations did not reveal CD‐specific signs of bowel lesions in Patient K. after 7 years of the disease onset, thus, testifying to the presence of isolated CD. The episode of intestinal manifestations of CD after 18 years of labial ulcerations was described elsewhere,^13^ which does not exclude the possibility of an inflammatory bowel process in Patient K. in the future. This necessitates regular endoscopic monitoring of intestinal mucosa in women with CD of the vulva for timely detection of lesions in the digestive tract.

The long diagnostic search with unsuccessful attempts of treatment using antimicrobial, anti‐inflammatory, immunomodulating, and other agents allowed to retrospectively reconstruct the development dynamics of isolated CD with perineal lesions. It is evident that the disease is characterized by the undulating course with a simultaneous or alternating involvement of the vulva and the perianal region. As the disease progresses, the recurrence rate increases, remission periods are reduced, and the absence of adequate therapy results in irreversible changes in the anatomy and physiology of the involved regions, which is primarily manifested by markedly pronounced bilateral asymmetric labial hypertrophy and induration.

Crohn's disease of the vulvar quires further studies, reports, and systematization of findings to develop diagnostic algorithms and therapeutic approaches to be used by clinicians in their routine practice.

## CONFLICT OF INTEREST

None declared.

## AUTHOR CONTRIBUTIONS

KRB: developed the theoretical framework, made a significant contribution to the concept and receipt of data, and data analysis and interpretation, provided data for Table 1, Figures 1, 2, and 3; took the lead in writing the manuscript, supervised the research. JED: aided in interpreting the results, participated in the compilation of the article and its critical processing for important intellectual content, co‐wrote the paper, and helped supervise the project. TAR: developed the theoretical framework, made a significant contribution to the concept of data, and data analysis and interpretation. NIN: performed biopsy; analyzed data and co‐wrote the paper. All authors discussed the results and implications and commented on the manuscript at all stages. All authors contributed extensively to the work presented in this paper. All authors reviewed the final manuscript.
